# Risk of neutropenia associated with Sacituzumab govitecan: a systematic review combined with the FAERS database and meta-analysis

**DOI:** 10.3389/fphar.2025.1714638

**Published:** 2026-01-12

**Authors:** Yin-Xue Xu, Qian Shen, Xiu-Fen Lu, Xiao-Lan Shen, Lei Zhang, Xi-Wen Qiao, Xue-Hui Zhang

**Affiliations:** Department of Pharmacy, the Affiliated Jiangsu Shengze Hospital of Nanjing Medical University, Suzhou, China

**Keywords:** FAERS, meta-analysis, neutropenia, pharmacovigilance analysis, Sacituzumab govitecan

## Abstract

**Objective:**

Sacituzumab govitecan (SG) has emerged as a therapeutic option for various cancers. Increasing reports of SG-associated neutropenia have attracted attention, emphasizing the need to fully characterize this risk.

**Methods:**

This study conducted a retrospective pharmacovigilance analysis using the Food and Drug Adverse Event Reporting System (FAERS) database. Cases of neutropenia associated with SG were extracted from the second quarter of 2020 through the second quarter of 2025 for disproportionality analysis. Additionally, a meta-analysis was performed on randomized controlled trials (RCTs) comparing SG (experimental group) with chemotherapy drugs (control group), retrieved from CNKI, Wanfang, VIP, PubMed, the Cochrane Library, EMBASE, and Web of Science.

**Results:**

In the FAERS, 500 cases of neutropenia were associated with SG treatment. Among antibody-drug conjugate (ADC) drugs, SG exhibited the strongest positive signal (reported odds ratio (ROR) = 17.91, 95% CI: 16.39–19.56). Subgroup analysis revealed that this signal widely existed in different gender, age, reporting groups and initial indications. Most events occurred within 30 days of initial initiation. Gender (10 days for males vs. 13 days for females, *P* < 0.05) and initial indications (12 days for breast cancer/lung cancer vs. 8 days for bladder cancer, *P* < 0.05) were significantly associated with time-to-onset. Five RCTs were included in this meta-analysis. The results showed that for all adverse event (AE) grades, SG is associated with an increased risk of neutropenia (odds ratio (OR) = 2.07, 95% CI: 1.09–3.93, *P* < 0.05). Subgroup analysis indicated that SG significantly increased the risk of all-grade neutropenia AE in patients with breast cancer (OR = 2.13, 95% CI: 1.68–2.69, *P* < 0.00001), and a similar trend was observed among bladder cancer patients (*P* < 0.00001). Although no significant difference emerged in the risk of all-grade AEs for lung cancer patients (*P* > 0.05), the control group exhibited a tendency toward higher risk, and the SG group demonstrated a significantly lower incidence of grade ≥3 AEs compared to controls (*P* < 0.05). In addition, the risk of neutropenia did not differ significantly between patients with the UGT1A1^*^1/^*^28 and UGT1A1^*^28/^*^28 genotypes compared to those with the UGT1A1^*^1/^*^1 genotype (*P* > 0.05).

**Conclusion:**

The combined analysis supports an elevated risk of neutropenia associated with SG. This AE exhibits significant cancer species specificity. Early intervention and management of neutropenia are therefore of considerable clinical importance.

## 1 Introduction

The first ADC received approval from the Food and Drug Administration (FDA) in 2000, catalyzing rapid advances in ADC applications for cancer therapy. These drugs combine highly specific targeting with potent cytotoxicity, positioning them among the most prominent areas of anticancer drug development (Fu et al., 2022). Sacituzumab govitecan (SG) is a representative ADC. It received accelerated approval from the FDA in April 2020 for treating patients with unresectable locally advanced or metastatic triple-negative breast cancer (TNBC) (Syed, 2020). This drug is the first ADC targeting trophoblast cell-surface antigen 2 (Trop-2) approved by the FDA for metastatic TNBC (Fu et al., 2022; Goldenberg et al., 2018; Rossi et al., 2024).

With the widespread use of SG, reports of associated AEs have increased. On 26 March 2025, the FDA issued a black box warning highlighting the risk of neutropenia linked to SG (FDA, 2025a). Notably, neutropenia appears to be a distinctive AE for SG compared to other ADC drugs, which needs to be paid high attention. Therefore, a comprehensive understanding of the incidence and risk profile of SG-related neutropenia is essential for healthcare providers.

Currently, evidence for drug safety evaluation is primarily derived from two sources: observational studies utilizing spontaneous reporting systems, such as FAERS, and meta-analyses based on RCTs. However, both methods exhibit inherent limitations. Spontaneous reporting systems, such as FAERS, excel at generating hypotheses from extensive real-world data and can rapidly identify potential safety signals (Fukazawa et al., 2018). Nevertheless, they suffer from reporting biases, difficulties in controlling confounding factors, and an inability to calculate true incidence rates, which often results in low signal specificity and prevents causal inference (FDA, 2025b). In contrast, meta-analysis provides the highest level of causal evidence by synthesizing data from multiple RCTs, serving as the gold standard for hypothesis confirmation (Guyatt et al., 1995). Yet its conclusions are constrained by the quantity and quality of available RCTs, and the strict inclusion criteria typical of RCTs may limit the generalizability of findings, making it difficult to detect rare or long-term AEs (Walker et al., 2008; Egger et al., 1997). Therefore, relying solely on any single method may fail to yield comprehensive or reliable conclusions: signals from FAERS alone can produce false positives, whereas meta-analysis might overlook potential risks absent from the analysis due to an insufficient number of RCTs. To address these limitations, we integrated real-world data mining of FAERS with a meta-analysis to comprehensively evaluate the association between SG and neutropenia AE.

Based on this foundation, this study aims to: 1. Use the FAERS database for data mining to preliminarily explore the correlation strength and signal distribution between SG and neutropenia AE; 2. Through systematic search and meta-analysis, the existing evidence of RCTs was quantitatively synthesized; 3. By comparing and integrating the results of the two methods, we can provide a higher level and more comprehensive evidence basis for guiding the safe clinical application of SG.

## 2 Materials and methods

### 2.1 Pharmacovigilance study

The pharmacovigilance analysis utilized all AE reports extracted from the FAERS database. ADCs were identified as the primary suspected drugs associated with neutropenia cases (Sacituzumab govitecan, Ado-trastuzumab Emtansined, Trastuzumab deruxtecan, Gemtuzumab ozogamicin, Brentuximab vedotin, Inotuzumab ozogamicin, Moxetumomab pasudotox, Polatuzumab vedotin, Enfortumab vedotin, Belantamab mafodotin, Loncastuximab tesirine, Tisotumab vedotin, Mirvetuximab soravtansin and Ibritumomab tiuxetan). AEs were coded according to the preferred terms (PTs) in Medical Dictionary of Regulatory Activities (MedDRA) (version 27.1). Neutropenia cases included PTs for autoimmune neutropenia, febrile neutropenia, neonatal neutropenia, neutropenic infection, and neutropenic sepsis. Following deduplication, the remaining neutropenia-related AE reports were evaluated to determine clinical characteristics such as gender, age, weight, country, reporting year, outcome, reporters type and other important clinical features.

Furthermore, we analyzed the proportional imbalance using four distinct algorithms: proportional reported odds ratio (PRR) and reported odds ratio (ROR), Bayesian confidence propagation neural network (BCPNN) and the multiple Gamma Poisson reduction method (MGPS) (Mytheen et al., 2023; Zhai et al., 2019). Table 1 shows the four-cell table of the ratio imbalance method. The four algorithms were summarized in Table 2, which were used to quantify the signal strength value representing the relevance between the interested drug and specific AE. If the criteria listed in Table 2 were met simultaneously, an AE would be considered highly associated with the treatment of the interested drug, with higher values indicating a more robust statistical correlation. Moreover, time-to-onset (TTO) data that was defined as the period of time from the initiation of the use of SG (START_DT) to the onset of AEs (EVENT_DT) were analyzed using the Weibull shape parameter (WSP) test. In order to ensure the accuracy of this calculation, reports with input errors (EVENT_DT earlier than START_DT), inaccurate date entries and missing specific data were excluded. The reasons for missing data are as follows: EVENT_DT and START_DT are not mandatory fields in FAERS reports; Reporters may omit or decline to provide this information. Some reports contain only the event year or employ an invalid date format, such as “2025″, “spring 2025″or “NA”, precluding accurate calculation. The incidence of AEs after the initiation of treatment depends on the drug mechanism of action and often varies over time. In contrast AEs that are not associated with drug treatment occur at a constant rate. The WSP test can determine the varying ratio of incidence of AEs. All analyses were performed using R software version 4.3.1 (The R Foundation for Statistical. Computing, Vienna. Austria).

**TABLE 1:** Four-cell table of proportional disequilibrium method.

Drugs	Target adverse reaction	Other adverse reactions	Total
Target drug	a	b	a+b
Other drugs	c	d	c + d
Total	a+c	b + d	N = a + b + c + d

**TABLE 2:** Four principal algorithms employed for signal detection.

Algorithms	Equation	Criteria
ROR	ROR = (a/c)/(b/d)	lower limit of 95% CI > 1, a ≥3
	95% CI = e^ln (ROR)±1.96 (1/a+1/b+1/c+1/d)^0.5^^	
PRR	PRR = [a/(a + b)]/[c/(c + d)]	PRR ≥2, χ^2^ ≥ 4, a ≥3
	χ^2^ = (ad − bc)^2^ (a + b + c + d)/[(a + b) (c + d) (a + c) (b + d)]	
	PRR 95% CI = e^ln (PRR)±1.96[(1/a-1/(a+b)+1/c −1/(c+d)]^0.5^^	
BCPNN	IC = log_2_^a(a+b+c+d)/[(a+c) (a+b)]^	IC025 > 0
	IC025 = e^ln (IC)−1.96(1/a+1/b+1/c+1/d)^0.5^^	
	95% CI = E (IC) ± 2V(IC)^0.5^	
EBGM	EBGM = a (a + b + c + d)/[(a+c) (a+b)]	EBGM05 > 2, a >0
	EBGM05 = e^ln (EBGM)±1.96(1/a+1/b+1/c+1/d)^0.5^^	

EBGM, empirical Bayesian geometric mean; IC, information component; E (IC), the IC, expectations; V (IC), the variance of IC; EBGM05, the lower limit of the 95% CI, of EBGM; 95% CI, 95% confidence interval.

### 2.2 Meta-analysis

The meta-analysis adhered to the 2020 Preferred Reporting Items for Systematic Reviews and Meta-Analyses (PRISMA) guidelines (Page et al., 2021). The details of the protocol are registered in the PROSPERO database (registration number CRD420251269064). [https://www.crd.york.ac.uk/PROSPERO/view/CRD420251269064]. We conducted a comprehensive search of CNKI, Wanfang database, VIP, PubMed, the Cochrane Library, EMBASE and Web of Science. Keywords included “Sacituzumab govitecan”, “Trodelvy”, “SG”, “cancer” and “neoplasms” among others, to identify eligible RCTs. This study established the inclusion criteria based on the PICO framework: cancer patients constituted the population (P), and treatment with SG defined the intervention (I). The comparison (C) was the chemotherapy regimen administered to the control group. The primary outcomes (O) included the incidence of SG-associated neutropenia and its relationship to UGT1A1 gene polymorphism.

The exclusion criteria were as follows: (1) Basic research, non-phase III RCTs, literature for which the full text could not be extracted, reviews, case reports and systematic reviews; (2) Literature lacking relevant outcome indicators.

Data collection was performed independently by two researchers, with any discrepancies resolved through discussion with a third researcher. The risk of bias for all included RCTs was assessed using version 5.1.0 of the Cochrane risk-of-bias tool, as recommended by the Cochrane Handbook for Systematic Reviews (The Cochrane Collbration, 2025). Meta-analyses were conducted using RevMan version 5.4.1. Odds ratios (OR) and 95% confidence intervals (CI) were calculated to evaluate the incidence of neutropenia. Heterogeneity among studies was assessed using the χ^2^ test (Hallajzadeh et al., 2019). A fixed-effect model was applied when *I* ^2^ < 50% and the *P* > 0.1, indicating low heterogeneity; Otherwise, a random-effect model was used. Sensitivity analysis was conducted through step-by-step exclusion of each study from the main analysis.

## 3 Result

### 3.1 Pharmacovigilance analysis

#### 3.1.1 Neutropenia AE signals for ADCs

**FIGURE 1:**
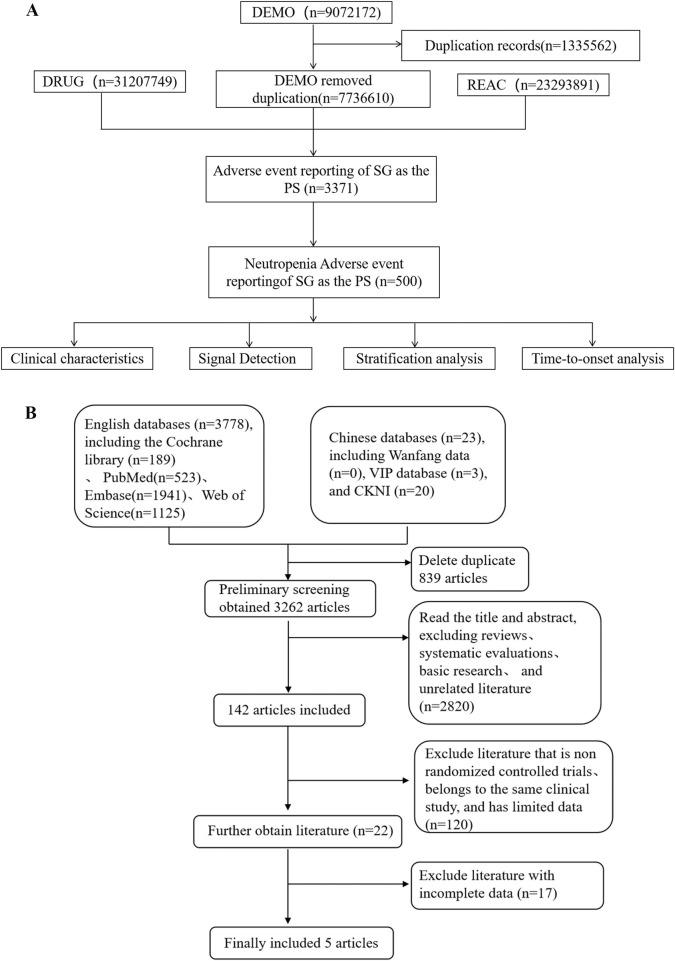
The flow diagrams of the safety analysis of neutropenia associated with SG in cancer patients. **(A)** Flowchart for screening neutropenia signals in FAERS from 2020Q2 to 2025Q2; **(B)** PRISMA flowchart summarizing the study selection process.

Figure 1A showed the screening process conducted on the FAERS database. We obtained AEs of SG from the FAERS database, totaling 7736610 cases. After deduplication and organization, we isolated 500 reports specifically related to neutropenia associated with SG. The AEs of other ADCs are also achieved using similar method.

**FIGURE 2:**
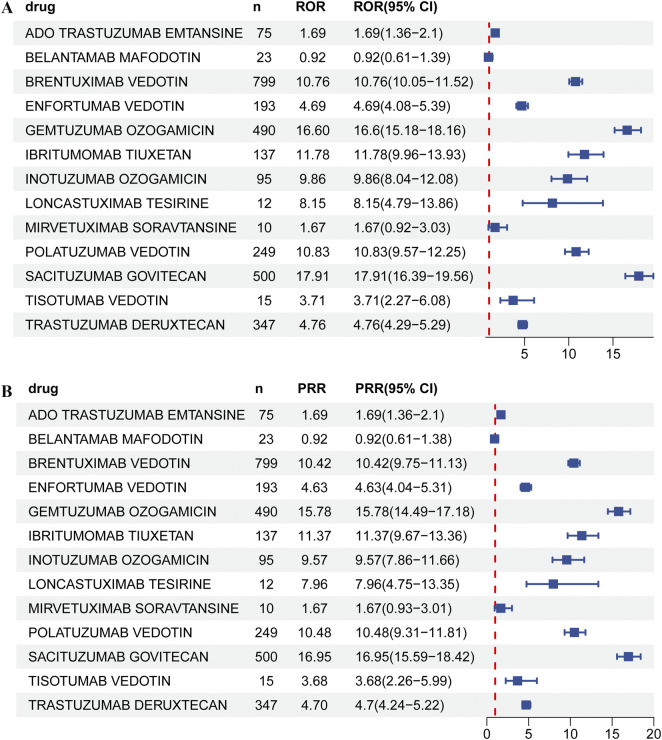
Neutropenia AE detection results for ADCs based on **(A)** ROR; **(B)** PRR.

The results of the disproportionality analysis of neutropenia AEs reported for different ADCs are presented in Figure 2 and Supplementary Table S1. Among the thirteen different ADCs investigated, neutropenia signals were detected for some drugs, but with SG exhibited the strongest and positive signal (ROR = 17.91 [95% CI: 16.39–19.56]; PRR (χ^2^) = 16.95 (7882.41); EBGM (EBGM05) = 16.9 (15.48); IC (IC025) = 4.08 (3.91)).

#### 3.1.2 Descriptive analysis

**FIGURE 3:**
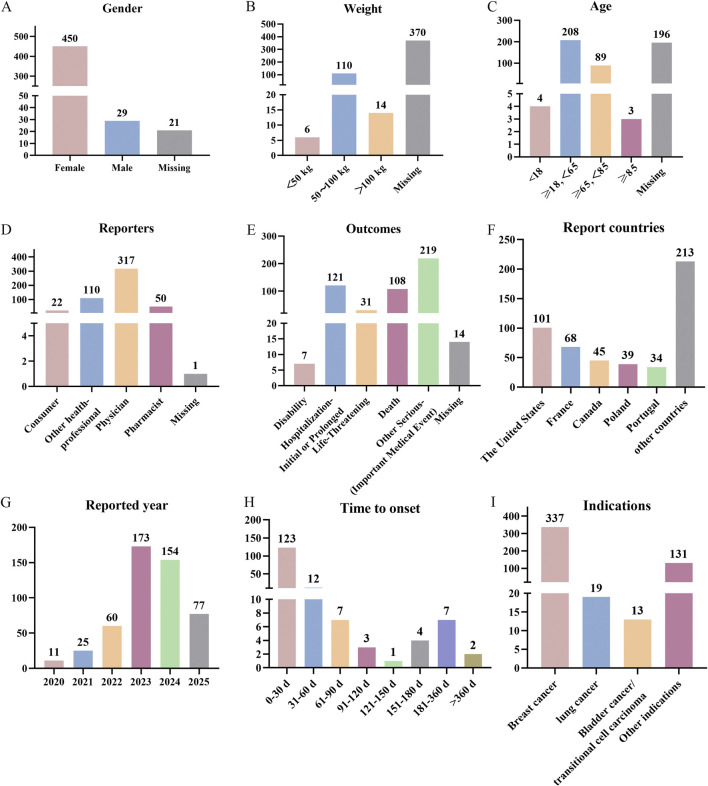
Overview of neutropenia AE reports related to SG in the FAERS database. **(A)** Gender distribution of patients. **(B)** Weight distribution of patients. **(C)** Age distribution of patients. **(D)** Occupational distribution of reporters. **(E)** Outcome distribution of patients. **(F)** Top 5 countries by the number of reports. **(G)** Annual number of reports. **(H)** Time to onset distribution of patients. **(I)** Indication distribution of patients.

Next, we described in detail the clinical characteristics associated with neutropenia cases (n = 500) in patients treated with SG, as summarized in Figure 3 and Supplementary Table S2. The results revealed that neutropenia is substantially more common in women (90.00%) than men (5.8%). Most patients weighed between 50 and 100 kg (22.0%). Regarding age, the 18–65 age group (17.8%) was the most affected. Physicians (63.4%) and other health-professional (22.0%) were the main reporters of neutropenia AE. Other serious (important medical event) was the most common outcome, occurring in 43.8% of cases, and 108 patients (21.6%) died from neutropenia. America reported the highest percentage (20.2%), followed by France (13.6%) and Canada (9.0%). Since the launch of SG in 2020, the number of reports has exhibited an overall upward trend, with most events reported in 2024 (30.8%). 159 (31.8%) of the cases did report the onset time of AEs. Most patients developed the disease in 0–30 days (77.4%), followed by 31–60 days (7.5%) and 61–90 days (4.4%). It was noteworthy that our findings suggested that AEs were most likely to occur in the early stages. Breast cancer patients constituted the largest proportion of cases (67.4%), followed by those with lung cancer (3.8%) and bladder cancer (2.6%).

#### 3.1.3 Subgroup analyses

**FIGURE 4:**
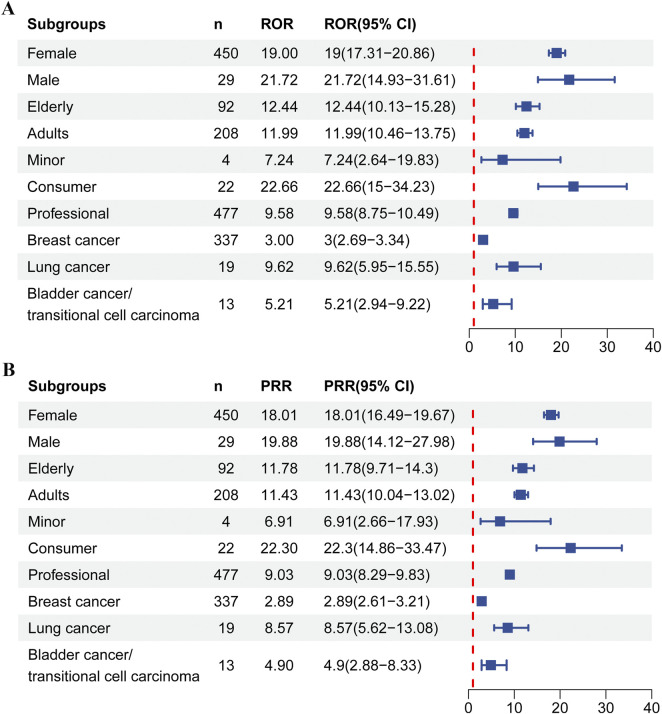
Neutropenia AE detection results for subgroups based on **(A)** ROR; **(B)** PRR. Elderly:≥65 years old; Adults: ≥18 and <65 years old; Minor:<18 years old.

To further assessed risk in specific populations, we conducted subgroup analyses. As shown in Figure 4 and Supplementary Table S3, these analyses revealed that the signal was widespread across demographic groups, although its strength and the reliability of the evidence varied. Among female patients, 450 reports yielded a strong positive signal (ROR = 19.00, 95% CI: 17.31–20.86). Male patients contributed 29 reports and also showed a positive signal (ROR = 21.72, 95% CI: 14.93–31.61). Medical professionals submitted 477 cases, for which a strong positive signal was detected (ROR = 9.58, 95% CI: 8.75–10.49). Consumers reported 22 cases and produced an even stronger signal (ROR = 22.66, 95% CI: 15.00–34.23). Strong and consistent positive signals emerged in both adult (n = 208, ROR = 11.99, 95% CI: 10.46–13.75) and elderly patients (n = 92, ROR = 12.44, 95% CI: 10.13–15.28). Only four reports were detected among minors, with a lower ROR and a wide confidence interval (ROR = 7.24, 95% CI: 2.64–19.83).

After stratification by tumor type, all predefined subgroups exhibited significant disproportionality signals. The lung cancer subgroup demonstrated the strongest association (ROR = 9.62, 95% CI: 5.95–15.55), despite a limited number of reported cases (n = 19). The bladder cancer subgroup ranked second (ROR = 5.21, 95% CI: 2.94–9.22, n = 13). The breast cancer subgroup contained the most reported cases (n = 337) and yielded an ROR of 3.00 (95% CI: 2.69–3.34).

#### 3.1.4 Time to onset (TTO)

**FIGURE 5:**
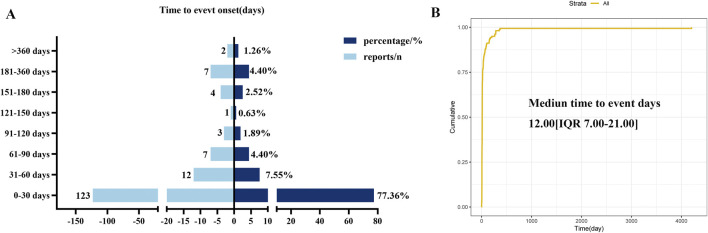
**(A)** Bar chart of time to onset of neutropenia AE. **(B)** Survival curve plot of AE onset time.

After excluding reports with incomplete data, we analyzed the TTO in 159 neutropenia patients. As shown in Figure 5B and Table 3, the median TTO was 12 days (Interquartile range [IQR]: 7–21 days). The majority of cases (n = 123, 77.36%) occurred within the first 30 days after initiating SG therapy. Over time, patients may adjust dosages or discontinue treatment due to AEs, leading to fewer reported cases (31–60 days: n = 12, 7.55%; 61–90 days: n = 7, 4.40%; 91–120 days: n = 3, 1.89%; 121–150 days: n = 1, 0.63%). The increased incidence between 151 and 180 days likely reflected the cumulative toxicity of SG observed in patients undergoing prolonged treatment. However, the overall frequency of AE progressively declined over time (Figure 5A).

**TABLE 3:** Weibull distribution of neutropenia AE.

Time to onset (days)			Weibull distribution		
Cases n	Media (IQR)	Min–max	Scale parameter (α 95% CI)	Shape parameter (β 95% CI)	Failure type
159	12 (7.00–21.00)	1–4204	31.59 (23.19–39.99)	0.62 (0.56–0.68)	Early failure

To further examine the temporal patterns of neutropenia AE risk, we analyzed the data using a Weibull distribution. The overall analysis showed a shape parameter (β) of 0.62, with an upper 95% CI of 0.68. The β value below one indicated an early failure-type model, with a decreasing hazard rate over time (Table 3), consistent with prior analytical results.

**FIGURE 6:**
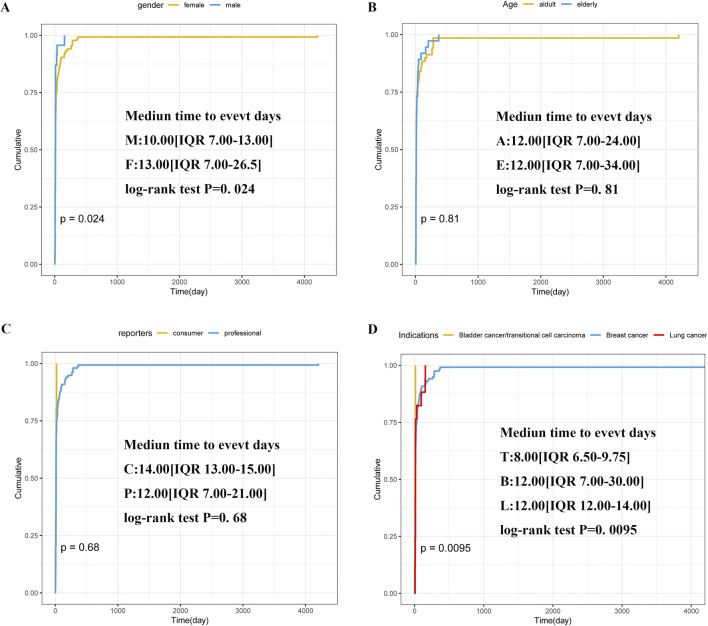
Subgroup analyses of TTO. **(A)** Gender; **(B)** Age; **(C)** Reporters; **(D)** Indications. Annotation: M(Male); F(female); E(Elderly):≥65 years old; A(Adults): ≥18 and <65 years old. C(Consumer); P(Professional); T(Transitional cell carcinoma); B(Breast cancer); L(Lung cancer).

Subsequently, we also discussed the influence of different genders, ages and reporters on TTO. In our analysis of subgroups, it was found that females (n = 135) exhibited a median event time of 13 days (IQR 7.00–26.50), compared to 10 days in males (n = 23) (IQR 7.00–13.00) (Figure 6A). Patients aged 18–65 (n = 69) showed a median neutropenia onset of 12 days (IQR 7.00–24.00), matching the 12-day median (IQR 7.00–34.00) observed in those over 65 (n = 37) (Figure 6B). Customers (n = 5) reported a median event time of 14 days (IQR 13.00–15.00), while health professionals (n = 154) documented a median of 12 days (IQR 7.00–21.00) (Figure 6C). The median time for bladder cancer/transitional cell carcinoma patients (n = 8) was 8 days (IQR 6.5–9.75), while breast (n = 120) and lung (n = 17) cancer patients both exhibited a median of 12 days, with IQR ranges of 7.00–30.00 and 12.00–14.00, respectively (Figure 6D).

### 3.2 Meta-analysis

#### 3.2.1 Characteristics of the studies

The security meta-analysis initially retrieved 3,801 records from various databases, of which five RCTs ultimately met the inclusion criteria (Rugo et al., 2023; Xu et al., 2024; Rugo et al., 2022; Powles et al., 2025; Paz-ares et al., 2024) (Figure 1B). Table 4 summarized the characteristics of the selected studies, which collectively enrolled 2,715 cancer patients. The SG treatment group comprised 1,358 participants, while 1,357 received placebo. As illustrated in Supplementary Figure S1, all included studies demonstrated low risk of bias.

**TABLE 4:** Characteristics of the studies included in the meta-analysis.

Author, year	NCT number	Indications	Sample size (n)		Outcomes	Intervention	
			Experiment	Control		Experiment	Control
Rugo et al. (2023)	NCT03901339	Breast cancer	272	271	①, ②	SG 10 mg/kg	Eribulin 1.4 or 1.23 mg/m^2^, vinorelbine 25 mg/m^2^, gemcitabine 800–∼1,200 mg/m^2^ or capecitabine 1,000∼1,250 mg/m^2^
Rugo et al. (2022)	NCT02574455	Breast cancer	267	262	①, ②	SG 10 mg/kg	Eribulin 1.4 or 1.23 mg/m^2^, vinorelbine 25 mg/m^2^, gemcitabine 800–∼1,200 mg/m^2^ or capecitabine 1,000∼1,250 mg/m^2^
Xu et al. (2024)	NCT04639986	Breast cancer	165	164	①, ②	SG 10 mg/kg	Eribulin 1.4 or 1.23 mg/m^2^, vinorelbine 25 mg/m^2^, gemcitabine 800–∼1,200 mg/m^2^ or capecitabine 1,000∼1,250 mg/m^2^
Powles et al. (2025)	NCT04527991	Urothelial carcinoma	355	356	①	SG 10 mg/kg	Paclitaxel 175 mg/m^2^, docetaxel 75 mg/m^2^ or vinflunine 320 mg/m^2^
Paz-Ares et al. (2024)	NCT05089734	Non-small cell lung cancer	299	304	①	SG 10 mg/kg	Docetaxel 75 mg/m^2^

Annotation, ①Neutropenia; ②Neutropenia associated with UGT1A1 gene polymorphis.

#### 3.2.2 Overall analysis

**FIGURE 7:**
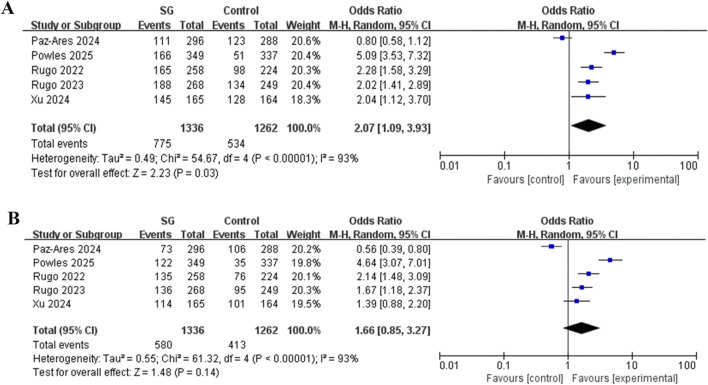
Meta analysis of the risk of neutropenia for SG. **(A)** Forest plot for neutropenia risk in all grades AE. **(B)** Forest plot for neutropenia risk in ≥3 grade AE.

Our study revealed that for all levels of AE, compared with the control group, the risk of neutropenia in SG group was significantly increased (OR = 2.07, 95% CI: 1.09–3.93, *P* < 0.05), with substantial heterogeneity observed between studies (*P* < 0.00001, *I* ^2^ = 93%) (Figure 7A). Moreover, for ≥3 grades AE, there was no significant difference in the risk of neutropenia between SG group and control group (OR = 1.66, 95% CI: 0.85–3.27, *P* > 0.05), and substantial heterogeneity was observed across the included studies (*P* < 0.00001, *I* ^2^ = 93%) (Figure 7B).

#### 3.2.3 Sensitivity analysis

For all-grade AE, the pooled analysis indicated that SG significantly increased the risk (OR = 2.07, *P* = 0.03). After excluding the study by Powles et al. (2025) (Powles et al., 2025), the OR decreased to 1.64 and lost statistical significance (*P* = 0.07). Following the exclusion of the Paz-Ares et al. (2024) (Paz-Ares et al., 2024), the OR increased to 2.67, a change that was highly significant (*P* < 0.0001).

For grade ≥3 AEs, the pooled analysis indicated no significant risk (OR = 1.66, *P* = 0.14). Excluding the Paz-Ares study (Paz-Ares et al., 2024), however, revealed a significant risk (OR = 2.19, *P* = 0.002). The subsequent removal of the Powles study (Powles et al., 2025) further reduced the effect size (OR = 1.29). All analyses exhibited high heterogeneity (*I* ^2^ ≥ 81%). Details are provided in Supplementary Table S4.

#### 3.2.4 Subgroups analysis

Subgroup analysis was conducted based on different indications.

**FIGURE 8:**
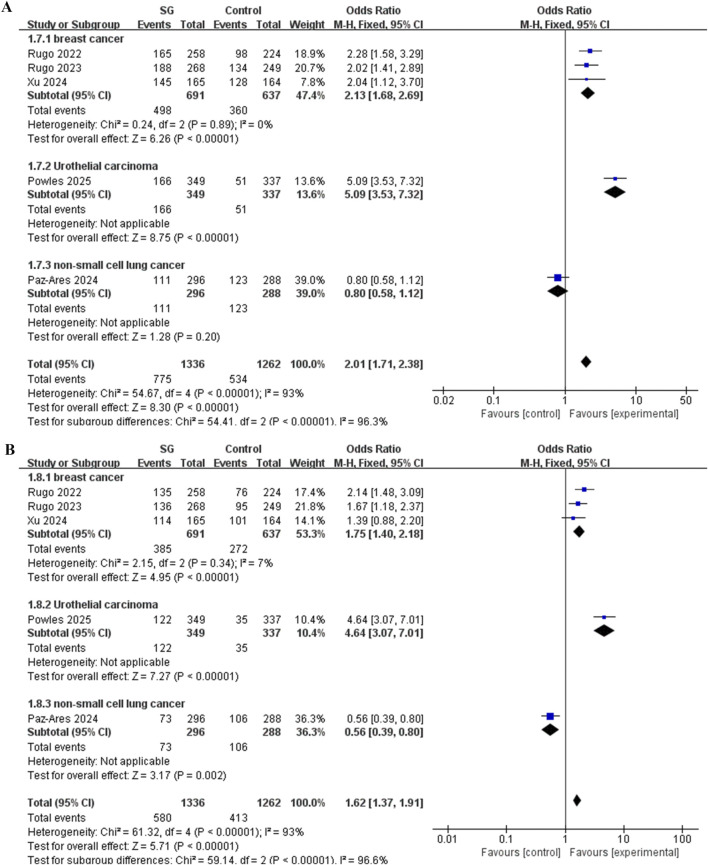
Forest plot for neutropenia risk of subgroups analysis. **(A)** All grades AE. **(B)** ≥3 grade AE.

The results indicated that for all-grade AE, breast cancer patients treated with SG exhibited a significantly elevated risk of neutropenia (OR = 2.13, 95% CI: 1.68–2.69, *P* < 0.00001). The single study (Powles et al., 2025) involving urothelial carcinoma patients found that SG significantly increased the risk of all-grade neutropenia AE compared with the control group (OR = 5.09, 95% CI: 3.53–7.32, *P* < 0.00001). Another single study (Paz-Ares et al., 2024) in non-small cell lung (NSCLC) cancer patients reported a lower risk of all-grade neutropenia AE in the SG group, though this difference was not statistically significant (OR = 0.8, 95% CI: 0.58–1.12, *P* > 0.05) (Figure 8A).

In the analysis of grade ≥3 AEs, SG significantly increased the risk of severe neutropenia among breast cancer patients (OR = 1.75, 95% CI: 1.40–2.18, *P* < 0.00001). The single urothelial carcinoma study (Powles et al., 2025) showed a markedly higher risk of grade ≥3 neutropenia AE with SG compared to controls (OR = 4.64, 95% CI: 3.07–7.01, *P* < 0.00001). The study (Paz-Ares et al., 2024) in NSCLC patients found that SG was associated with a significantly reduced risk of grade ≥3 neutropenia AE (OR = 0.56, 95% CI: 0.39–0.80, *P* < 0.05) (Figure 8B).

Patients homozygous for the UGT1A1^*^28 allele have been reported to develop severe neutropenia following SG treatment (Baek et al., 2022); Moreover, the incidence of neutropenia of all grades increases with the number of variant ^*^28 alleles (wild-type ^*^1/^*^1 < heterozygous ^*^1/^*^28 < homozygous ^*^28/^*^28) (Bardia et al., 2019). Accordingly, this study also examined the association between UGT1A1 genotype and neutropenia. The results are presented in Table 5. There was no significant difference in neutropenia risk between UGT1A1^*^1/^*^28 and UGT1A1^*^28/^*^28 patients compared with UGT1A1^*^1/^*^1 patients, regardless of all grades or AE ≥ 3 grades (*P* > 0.05).

**TABLE 5:** Association between UGT1A1 gene polymorphism and neutropenia.

Subgroups	n	Heterogeneity		Statistical analysis	
		I^2^/%	*P*	OR (95% CI)	*P*
^*^1/^*^28 vs.^*^1/^*^1					
All grades	3 (Rugo et al., 2023; Xu et al., 2024; Rugo et al., 2022)	47	0.15	0.92 (0.62, 1.36)	0.67
≥3 grade	3 (Rugo et al., 2023; Xu et al., 2024; Rugo et al., 2022)	52	0.12	1.23 (0.69, 2.18)	0.48
^*^28/^*^28 vs.^*^1/^*^1					
All grades	2 (Rugo et al., 2023; Rugo et al., 2022)	0	0.87	1.22 (0.64, 2.33)	0.54
≥3 grade	2 (Rugo et al., 2023; Rugo et al., 2022)	0	0.36	1.60 (0.89, 2.88)	0.12

## 4 Discussion

In 2025, the FDA issued a warning regarding the risk of severe neutropenia in patients undergoing SG treatment. With the extensive application of SG in oncology, reports have indicated an association with severe neutropenia as an AE (Baek et al., 2022; Prescott et al., 2022). Another study reported that 13% of patients who experienced this AE exhibited grade 4 severity, with an incidence reaching 8% in those with pretreated metastatic TNBC (Bardia et al., 2019; Bardia et al., 2021). Severe hematological AEs can result in bleeding, febrile neutropenia, and other complications if left untreated. Consequently, the relationship between SG and neutropenia warrants closer investigation.

This study identified the strongest correlation signal between SG and neutropenia AE among various related drugs through a signal mining analysis of the FAERS database (ROR = 17.91 [95% CI: 16.39–19.56], PRR = 16.95 (χ^2^ = 7882.41), EBGM = 16.9 (EBGM05: 15.48), IC = 4.08 (IC025: 3.91)). A subsequent meta-analysis of RCTs further confirmed that patients receiving SG exhibited a significantly higher risk of all-grade neutropenia AE compared with the control group (OR = 2.07, 95% CI: 1.09–3.93). The signal detection results from the spontaneous reporting system aligned closely with those of the meta-analysis of RCTs data, indicating that clinicians should remain vigilant for neutropenia AE when prescribing SG. Necessary monitoring and patient education are advised, including guidance on recognizing early symptoms of AEs and the importance of timely reporting.

In addition to confirming the correlation strength and causal relationship between SG and neutropenia AE, our study further revealed the time characteristics of the AE. In our study, neutropenia occurred predominantly (77.36% of cases) within 30 days following the initiation of SG treatment. The median TTO was 12 days, consistent with an early failure pattern (Figure 5; Table 3). This implies the importance of neutropenia level monitoring early during SG treatment, which helps minimize the risk of drug-induced neutropenia and ensures patient safety. Next, we conducted a subgroup analysis to evaluate the TTO and examined how gender, age, reporter type, and initial indications influence the timing of neutropenia induced by SG. The results indicated that patient gender and initial indications were statistically significantly associated with TTO, whereas age and reporter type showed no statistically significant effects. This study found that the median TTO of male patients (10.0 days) was significantly shorter than that of female patients (13.0 days) (*P* < 0.05, Figure 6A). These results indicated that closer monitoring of male patients may be warranted during the clinical use of SG, particularly within the first 2 weeks of treatment. The same statistical significance was observed in the indication subgroup. Notably, patients with bladder cancer exhibited the shortest median TTO (8.0 days), which was significantly shorter than that of patients with breast or lung cancer (both 12.0 days, *P* < 0.05, Figure 6D). Patients with bladder cancer typically present with underlying urinary system dysfunction. The SG antibody, a monoclonal antibody targeting Trop-2, is primarily cleared through renal excretion after metabolism. This pharmacokinetic profile may lead to its higher concentration and prolonged retention in the bladder, potentially enabling it to reach the threshold for inducing neutropenia more rapidly (Ocean et al., 2017). This exploratory analysis suggested that the TTO of SG-induced neutropenia may differ by patient gender and initial indications, with bladder cancer patients potentially experiencing a more rapid onset. However, given the imbalanced sample sizes, these findings should be considered hypothesis-generating rather than conclusive. Further validation in large prospective cohorts or RCTs is needed to clarify the influence of gender and specific indications (particularly bladder cancer) on the time to this AE.

Sensitivity analysis revealed that the overall association between SG and neutropenia risk was not robust in this meta-analysis, as the overall pooled OR and its statistical significance proved highly sensitive to the exclusion of individual studies. Specifically, after excluding the study by Powles et al. (2025), (urothelial carcinoma), the OR was significantly reduced and lost statistical significance; After excluding the study by Paz-Ares et al. (2024), (NSCLC), the OR increased substantially. This result indicates significant heterogeneity among the studies, likely attributable to differences in the treatment indications (cancer types) of the enrolled patients. Therefore, the overall pooled OR (OR = 2.07) is less clinically reliable due to the high heterogeneity. To systematically test this hypothesis and identify the specific source of heterogeneity, we performed a subgroup analysis based on treatment indication (breast cancer, NSCLC, urothelial carcinoma).

Subgroup analysis of the meta-analysis revealed that the risk of neutropenia associated with SG was highly dependent on tumor type. In the ungrouped analysis, the overall risk of grade 3 or higher AE did not reach statistical significance (*P* > 0.05), and substantial heterogeneity was observed (Figure 7B). Stratification by indications reduced heterogeneity within each subgroup to zero, indicating that indication type was the principal source of between-study variation (Figure 8). More importantly, after stratification, in the breast cancer subgroup, SG significantly increased the risk of grade ≥3 AE (*P* < 0.00001). These results illustrated that safety evaluations which overlook disease context may yield misleading conclusions. For breast cancer patients, the results clearly indicated that SG significantly increased the risk of neutropenia AE across all severity grades, particularly severe reactions of grade 3 or higher, which warranted heightened vigilance. Clinicians should thoroughly inform patients of this risk when prescribing SG and enhance monitoring and management accordingly. In our study, there was only one available study for patients with urothelial carcinoma (Powles et al., 2025). This study strongly suggested that SG also increases the risk of neutropenia-related AE in this population (*P* < 0.00001), including events of grade 3 or higher (*P* < 0.00001). However, since this conclusion derived from a single study, its generalizability requires validation through additional clinical trials in urothelial carcinoma patients. For patients with NSCLC, the findings are particularly distinctive and noteworthy. Although no significant difference was observed in the risk of all-grade neutropenia AE (*P* > 0.05), a trend toward higher risk was evident in the control group. Most notably, for grade 3 or higher AEs, the only available study (Paz-Ares et al., 2024) indicated a significantly lower risk in the SG group compared to the control group (*P* < 0.05). In clinical trials across different cancer types, the control group treatments may be different. For instance, in the NSCLC study (Paz-Ares et al., 2024), the drug used in the control group was docetaxel. From the clinical data, the risk of neutropenia caused by docetaxel was higher than that caused by SG (in EVOKE-01 study (Paz-Ares et al., 2024), the grade 3–4 neutropenia rate of docetaxel was 36.8%, while that of SG was 24.7%), suggesting that in the second-line or later treatment of NSCLC, SG presents a relatively lower risk of neutropenia than the standard chemotherapeutic agent docetaxel. This finding contrasted sharply with observations in breast and urothelial carcinoma, suggesting the impact of different indications on drug safety. Clinicians should therefore consider the specific indication when prescribing SG and adopt distinct risk assessment, patient education, and monitoring strategies for different cancers.

In the FAERS subgroup analysis, the correlation signal between SG and neutropenia was observed across all gender, age, reporter and indication subgroups, suggesting that the associated risk may be universal rather than limited to specific populations (Figure 4). The core finding of this study is that the signal demonstrated high reliability in both adult and elderly patients (adult: ROR = 11.99, 95% CI: 10.46–13.75; elderly: ROR = 12.44, 95% CI: 10.13–15.28). In the gender-based subgroup analysis, female patients constituted the vast majority of reports (90.00%), reflecting the primary indication for SG (TNBC) in this population. While the reported signal strengths were similar between women and men (ROR: 19.00 vs. 21.72), this distribution is likely attributable to indication bias rather than reliable evidence of a biological risk difference. The number of male case reports was limited (n = 29) with a wide CI. The slightly higher point estimate for men (ROR = 19.00) suggests they may face a non-negligible risk in other SG indications, such as bladder cancer, though this requires confirmation with more data. Consequently, FAERS data does not reliably support a higher biological risk in women. Moreover, the ROR value reported by consumers is 2.4 times that of professionals, and the non-overlapping CI strongly suggested the presence of reporting bias, highlighting potential risks specific to non-medical monitoring environments. Currently, data for the pediatric population remained extremely limited, emphasizing an urgent need for additional post-marketing surveillance. Regulatory agencies, healthcare providers, and patients should be made aware of this potential risk.

By integrating the subgroup analyses of indications from both the meta-analysis and the FAERS database, this study provides a comprehensive evaluation of the risk of neutropenia associated with SG across different cancer types. The risk of neutropenia AE induced by SG was found to be highly cancer-specific. SG should not be categorically classified as “safe” or “unsafe”, but must instead be evaluated in conjunction with specific tumor types and clinical background. Notably, heterogeneity was observed between results from FAERS and meta-analyses across different cancer types. Especially in patients with lung cancer, the FAERS data revealed a strong distribution signal (ROR = 9.62, 95% CI: 5.95–15.55, Figure 4). However, the meta-analysis of RCTs did not confirm this risk (all grades: OR = 0.8, 95% CI: 0.58–1.12, *P* > 0.05, Figure 8A; ≥3 grade: OR = 0.56, 95% CI: 0.39–0.80, *P* < 0.05; Figure 8B). This discrepancy likely stemmed from the inherent reporting bias associated with spontaneous reporting systems. SG is commonly used to treat lung cancer; However, neutropenia is a known AE that clinicians closely monitor during treatment, resulting in a significantly higher reporting rate than other AEs. This difference may also be due to the limited cohort of lung cancer patients treated with SG, which amplifies the statistical effect of individual case reports. Therefore, the high ROR value for lung cancer in FAERS should be interpreted as a signal worthy of attention rather than a confirmed risk. The meta-analysis provides higher-level evidence that the risk did not increase significantly under the controlled test conditions. These results suggested that SG may be a preferable alternative to chemotherapy (such as docetaxel), for patients particularly sensitive to neutropenia-related AEs like hematologic toxicity. Therefore, for patients with lung cancer, SG can be regarded as a more controllable option compared with traditional chemotherapy. In contrast, for patients with breast cancer, both the FAERS signal (ROR = 3.00, 95% CI: 2.69–3.34, Figure 4) and the meta-analysis results (all grades: OR = 2.13, 95% CI: 1.68–2.69, *P* < 0.00001, Figure 8A; ≥3 grade: OR = 1.75, 95% CI: 1.40–2.18, *P* < 0.00001; Figure 8B) demonstrated a highly consistent and statistically significant association, strongly supporting a causal link between SG and neutropenia AE in this population. These findings suggested that clinicians should implement routine and proactive prevention and monitoring strategies for breast cancer patients receiving SG to mitigate these AEs. Bladder cancer data exhibited a similar trend. In our study, although FAERS reports were limited and only one RCT was available, its high-quality evidence warrants clinical attention to this risk in bladder cancer patients. This study highlights the value of combining multiple evidence sources for drug safety assessment. As an efficient hypothesis-generation tool, FAERS can rapidly identify potential safety signals (such as lung and bladder cancer), but these signals require confirmation through well-designed hypothesis-testing studies, such as RCTs or their meta-analyses.

SG comprises three components: Trop-2, the active irinotecan metabolite SN-38, and SN-38 connector (Goldenberg et al., 2018; Sharkey et al., 2015; Golden et al., 2015; Cardillo et al., 2015; Cardillo et al., 2011). SN-38 is mainly metabolized into inactive SN-38G via UGT1A1. When the gene encoding UGT1A1 is mutated and the enzyme activity is reduced, SN-38 accumulates *in vivo* and causes toxic and side effects (Hulshof et al., 2023). Studies have identified polymorphisms in the UGT1A1 gene as a major risk factor for neutropenia (Karas and Innocenti, 2022; Nelson et al., 2021). Contrary to these findings, our meta-analysis did not identify a significant association between the UGT1A1^*^28 allele and the risk of SG-induced neutropenia (*P* > 0.05, Table 5). This result must be interpreted with considerable caution, because this analysis is only based on 2-3 studies, with small sample size and lack of statistical power, creating a high risk of type II error. Consequently, the present findings are insufficient to refute prior evidence but instead indicate that larger-scale studies are required to reach a definitive conclusion. Because the proportion of the ^*^28 homozygous genotype in the population is relatively low (Jada et al., 2007), and SG does not increase the withdrawal rate, these AEs can be managed clinically. Consequently, patients receiving SG treatment do not require routine UGT1A1 gene testing prior to treatment, nor do they need initial dose adjustments based on UGT1A1 genotype. However, patients identified as UGT1A1^*^28 homozygous should be closely monitored.

Although this study provided multidimensional insights into the association between SG and neutropenia AE by integrating real-world data and evidence from RCTs, there are still some inherent limitations.

In pharmacovigilance analysis, the FAERS database relies on spontaneous reporting, which suffers from inherent limitations such as incomplete report, an inability to confirm causality, lack of exposed population denominator and multiple potential biases including reporting bias and media influence (Fukazawa et al., 2018; FDA, 2025b). Second, subgroup analyses, especially those involving small sample sizes, such as men, consumers, minors, lung cancer, or bladder cancer, should be interpreted as exploratory findings, and their underlying mechanisms, such as the pharmacokinetics of SG across gender and disease states, require validation through future prospective or enrichment-design studies. In addition, although the overall *P*-value in the subgroup analysis of TTO indications was significant, the sample sizes were uneven (the n of bladder cancer was 8), which should be interpreted carefully. Furthermore, the TTO analysis in this study was restricted to 159 cases (31.8%) with complete date information, excluding over two-thirds of cases lacking a clear date. This approach may introduce selection bias, as reports with accurate dates could systematically originate from specific reporter types, such as professional medical institutions rather than consumers, or reflect events of a particular severity or level of concern. Consequently, extending this conclusion to all patients using SG who develop neutropenia therefore warrants particular caution. Moreover, a significant limitation of this FAERS analysis is the high proportion of missing demographic data. Specifically, weight information was unreported in up to 74.0% of cases, while age information was missing in 39.2%. Finally, FAERS cannot provide the exact total number of drug users, so we can only calculate the ROR value, which reflects the reporting distribution, rather than the true incidence of AEs as in RCTs. This limitation constrains our assessment of the absolute risk magnitude.

In meta-analysis, RCTs typically adhere to strict inclusion criteria and often involve highly screened, homogeneous populations, which may not adequately represent the diverse and complex patient groups receiving SG in real-world settings (Walker et al., 2008; Egger et al., 1997). Second, although we merged the breast cancer studies, differences may persist across the included RCTs in trial design, control group selection, baseline patient characteristics, and AE definitions. Such heterogeneity could influence the interpretation of the pooled results. In addition, a major limitation of this study is the substantial statistical heterogeneity (*I* ^2^ = 93%) observed across the included studies. While sensitivity and subgroup analyses identified “cancer type” as a significant source of this heterogeneity, considerable residual heterogeneity remained. Consequently, the overall pooled OR (OR = 2.07) derived from this meta-analysis should be considered exploratory, and its direct extrapolation or reliability for guiding specific clinical decisions is limited. Finally, the number of RCTs included in this analysis is limited, particularly in the assessment of the correlation between UGT1A1 gene polymorphism and neutropenia, where only one study was available for both the NSCLC and urothelial carcinoma subgroups. This limits our ability to conduct combined analysis or assess publication bias. The robustness of the conclusions therefore depends heavily on the quality of these individual studies, underscoring the need for more high-quality research to strengthen the evidence base.

## 5 Conclusion

In conclusion, by integrating data mining of FAERS with meta-analysis of RCTs, this study provided combined evidence to support an elevated risk of neutropenia associated with SG, and exhibited marked cancer type specificity in its association with this AE. As an efficient “signal detection radar”, FAERS identified potential safety signals (such as strong signals in lung and bladder cancer subgroups) from massive real-world data. Meanwhile, meta-analysis played the role of “precision verification tool”, using data of rigorously designed RCTs to either confirm or refute the causality of these signals, as exemplified by the confirmation of breast cancer risk and the questioning of lung cancer risk. This combination substantially enhanced the efficiency and reliability of drug safety investigations. Researchers and clinicians should consider this association. Future studies could explore the underlying biological mechanisms and validate the findings in the real world.

## Data Availability

The original contributions presented in the study are included in the article/Supplementary Material, further inquiries can be directed to the corresponding author.
