# Supplementary material for: Risk of neutropenia associated with Sacituzumab govitecan: a systematic review combined with the FAERS database and meta-analysis
Source: Front Pharmacol. 2026 Jan 12;16:1714638. doi: 10.3389/fphar.2025.1714638 (PMC12832817; doi:10.3389/fphar.2025.1714638)
Supplement: Supplementary file 1 [file DataSheet1.docx]

**Supplementary Table 1**. Signal detection results of 13 ADCs associated neutropenia AE

| Drugs | n | ROR（95%CI） | PRR(χ^2^) | EBGM(EBGM05) | IC(IC025) |
| --- | --- | --- | --- | --- | --- |
| ADO TRASTUZUMAB EMTANSINE | 75 | 1.69 ( 1.36 - 2.1 ) | 1.69 ( 22.75 ) | 1.69 ( 1.36 ) | 0.75 ( 0.42 ) |
| BELANTAMAB MAFODOTIN | 23 | 0.92 ( 0.61 - 1.39 ) | 0.92 ( 0.16 ) | 0.92 ( 0.61 ) | -0.12 ( -0.71 ) |
| BRENTUXIMAB VEDOTIN | 799 | 10.76 ( 10.05 - 11.52 ) | 10.42 ( 7221.8 ) | 10.38 ( 9.69 ) | 3.38 ( 3.26 ) |
| ENFORTUMAB VEDOTIN | 193 | 4.69 ( 4.08 - 5.39 ) | 4.63 ( 573.73 ) | 4.63 ( 4.03 ) | 2.21 ( 1.98 ) |
| GEMTUZUMAB OZOGAMICIN | 490 | 16.6 ( 15.18 - 18.16 ) | 15.78 ( 6967.15 ) | 15.74 ( 14.39 ) | 3.98 ( 3.8 ) |
| IBRITUMOMAB TIUXETAN | 137 | 11.78 ( 9.96 - 13.93 ) | 11.37 ( 1346.17 ) | 11.36 ( 9.6 ) | 3.51 ( 3.16 ) |
| INOTUZUMAB OZOGAMICIN | 95 | 9.86 ( 8.04 - 12.08 ) | 9.57 ( 739.21 ) | 9.57 ( 7.81 ) | 3.26 ( 2.84 ) |
| LONCASTUXIMAB TESIRINE | 12 | 8.15 ( 4.79 - 13.86 ) | 7.96 ( 85.48 ) | 7.96 ( 4.68 ) | 2.99 ( 1.69 ) |
| MIRVETUXIMAB SORAVTANSINE | 10 | 1.67 ( 0.92 - 3.03 ) | 1.67 ( 2.96 ) | 1.67 ( 0.92 ) | 0.74 ( -0.18 ) |
| POLATUZUMAB VEDOTIN | 249 | 10.83 ( 9.57 - 12.25 ) | 10.48 ( 2252.32 ) | 10.47 ( 9.26 ) | 3.39 ( 3.16 ) |
| SACITUZUMAB GOVITECAN | 500 | 17.91 ( 16.39 - 19.56 ) | 16.95 ( 7882.41 ) | 16.9 ( 15.48 ) | 4.08 ( 3.91 ) |
| TISOTUMAB VEDOTIN | 15 | 3.71 ( 2.27 - 6.08 ) | 3.68 ( 31.33 ) | 3.68 ( 2.25 ) | 1.88 ( 0.96 ) |
| TRASTUZUMAB DERUXTECAN | 347 | 4.76 ( 4.29 - 5.29 ) | 4.7 ( 1028.26 ) | 4.7 ( 4.23 ) | 2.23 ( 2.06 ) |

**Supplementary Table 2**. Clinical characteristics of neutropenia AE with SG from the FAERS database (2020 Q2 to 2025 Q2)

| **Characteristics** | **Case Number,n** | **Case Proportion, %** |
| --- | --- | --- |
| **Gender** |  |  |
| Female | 450 | 90.0% |
| Male | 29 | 5.8% |
| Missing | 21 | 4.2% |
| **Weight** |  |  |
| ＜50 kg | 6 | 1.2% |
| 50～100 kg | 110 | 22.0% |
| ＞100 kg | 14 | 2.8% |
| Missing | 370 | 74.0% |
| **Age（Years）** |  |  |
| <18 | 4 | 0.8% |
| ≥18，＜65 | 208 | 41.6% |
| ≥65，＜85 | 89 | 17.8% |
| ≥85 | 3 | 0.6% |
| Missing | 196 | 39.2% |
| **Reporters** |  |  |
| Consumer | 22 | 4.4% |
| Other health-professional | 110 | 22.0% |
| Physician | 317 | 63.4% |
| Missing | 1 | 0.2% |
| Pharmacist | 50 | 10.0% |
| **Outcomes** |  |  |
| Death | 108 | 21.6% |
| Disability | 7 | 1.4% |
| Hospitalization-Initial or Prolonged | 121 | 24.2% |
| Life-Threatening | 31 | 6.2% |
| Other Serious (Important Medical Event) | 219 | 43.8% |
| Missing | 14 | 2.8% |
| **Report countries** |  |  |
| The United States | 101 | 20.2% |
| France | 68 | 13.6% |
| Canada | 45 | 9.0% |
| Poland | 39 | 7.8% |
| Portugal | 34 | 6.8% |
| other countries | 213 | 42.6% |
| **Reported year** |  |  |
| 2025 | 77 | 15.4% |
| 2024 | 154 | 30.8% |
| 2023 | 173 | 34.6% |
| 2022 | 60 | 12.0% |
| 2021 | 25 | 5.0% |
| 2020 | 11 | 2.2% |
| **Time to onset(n=159)** |  |  |
| 0-30 d | 123 | 77.4% |
| 31-60 d | 12 | 7.5% |
| 61-90 d | 7 | 4.4% |
| 91-120 d | 3 | 1.9% |
| 121-150 d | 1 | 0.6% |
| 151-180 d | 4 | 2.5% |
| 181-360 d | 7 | 4.4% |
| >360 d | 2 | 1.3% |
| **Indications** |  |  |
| Breast cancer | 337 | 67.4% |
| lung cancer | 19 | 3.8% |
| Bladder cancer/transitional cell carcinoma | 13 | 2.6% |
| Other indications | 131 | 26.2% |

**Supplementary Table 3**. Signal detection results of subgroups associated neutropenia AE

| Subgroups | n | ROR（95%CI） | PRR(χ^2^) | EBGM(EBGM05) | IC(IC025) |
| --- | --- | --- | --- | --- | --- |
| Female | 450 | 19 ( 17.31 - 20.86 ) | 18.01 ( 7528.97 ) | 17.8 ( 16.22 ) | 4.15 ( 3.97 ) |
| Male | 29 | 21.72 ( 14.93 - 31.61 ) | 19.88 ( 539.81 ) | 19.86 ( 13.65 ) | 4.31 ( 3.08 ) |
| Elderly | 92 | 12.44 ( 10.13 - 15.28 ) | 11.78 ( 957.89 ) | 11.74 ( 9.56 ) | 3.55 ( 3.1 ) |
| Adults | 208 | 11.99 ( 10.46 - 13.75 ) | 11.43 ( 2062.29 ) | 11.37 ( 9.91 ) | 3.51 ( 3.24 ) |
| Minor | 4 | 7.24 ( 2.64 - 19.83 ) | 6.91 ( 20.36 ) | 6.9 ( 2.52 ) | 2.79 ( 0.32 ) |
| Consumer | 22 | 22.66 ( 15 - 34.23 ) | 22.3 ( 466.88 ) | 22.24 ( 14.72 ) | 4.47 ( 2.96 ) |
| Professional | 277 | 9.58 ( 8.75 - 10.49 ) | 9.03 ( 3580.29 ) | 8.98 ( 8.2 ) | 3.17 ( 3.01 ) |
| Breast cancer | 337 | 3 ( 2.69 - 3.34 ) | 2.89 ( 434.6 ) | 2.83 ( 2.54 ) | 1.5 ( 1.33 ) |
| Lung cancer | 19 | 9.62 ( 5.95 - 15.55 ) | 8.57 ( 128.34 ) | 8.54 ( 5.28 ) | 3.09 ( 1.94 ) |
| Bladder cancer/ transitional cell carcinoma | 13 | 5.21 ( 2.94 - 9.22 ) | 4.9 ( 39.97 ) | 4.8 ( 2.71 ) | 2.26 ( 1.1 ) |

**Supplementary Table 4**. Summary of sensitivity analysis results

|  | all-grade AE | | | grade ≥3 AEs | | |
| --- | --- | --- | --- | --- | --- | --- |
|  | **OR**[**95% CI**] | ***P*** | **I^2^** | **OR**[**95% CI**] | ***P*** | **I^2^** |
| **Include all studies** | 2.07[1.09, 3.93] | 0.03 | 93% | 1.66[0.85, 3.27] | 0.14 | 93% |
| Exclud **Paz-Ares 2024** | **2.67[1.67, 4.25]** | **<0.0001** | 81% | **2.19[1.34, 3.59]** | **0.002** | 84% |
| Exclud **Powles 2025** | **1.64[0.96, 2.80]** | **0.07** | 86% | 1.29[0.71, 2.36] | 0.41 | 90% |
| Exclud **Rugo 2022** | 2.02[0.88, 4.66] | 0.10 | 94% | 1.56[0.66, 3.70] | 0.31 | 95% |
| Exclud **Rugo 2023** | 2.09[0.90, 4.85] | 0.09 | 95% | 1.67[0.68, 4.07] | 0.26 | 95% |
| Exclud **Xu 2024** | 2.08[0.97, 4.44] | 0.06 | 95% | 1.74[0.75, 4.01] | 0.19 | 95% |


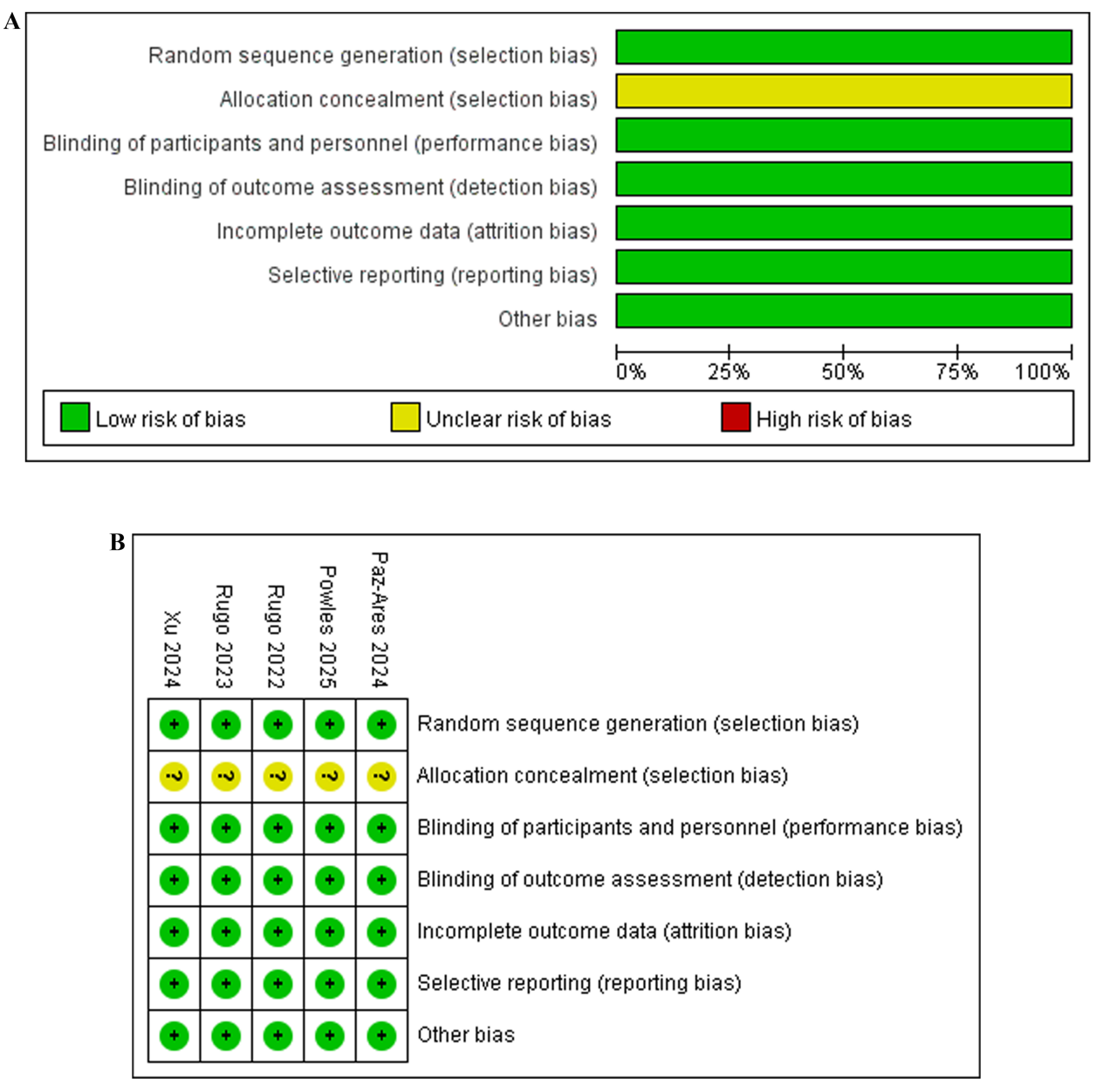


**Supplementary Figure 1**. Assessment of bias. (A) Bias risk bar chart; (B) Bias risk map
